# Isometric Tunnel Placement in Ulnar Collateral Ligament Reconstruction with Single CT Scan

**DOI:** 10.1155/2017/5128639

**Published:** 2017-10-18

**Authors:** Erica Kholinne, Rizki Fajar Zulkarnain, Arnold Adikrishna, Bin Zhu, Han Pyo Hong, In-Ho Jeon

**Affiliations:** ^1^Department of Orthopedic Surgery, Asan Medical Center, University of Ulsan College of Medicine, Seoul, Republic of Korea; ^2^Department of Orthopedic Surgery, St. Carolus Hospital, Jakarta, Indonesia

## Abstract

**Background:**

Isometric tunnel placement for anterior bundle of the medial collateral ligament (MCL) reconstruction is mandatory for successful surgery.

**Purpose:**

This study aimed to demonstrate a useful method for identifying isometric tunnel placement using a single computed tomography (CT) scan.

**Study Design:**

Descriptive Laboratory Study.

**Methods:**

Five normal elbows were scanned at 4 different flexion angles at 45° increment. Three-dimensional models were analyzed using 2 different approaches: single and multiple CT scans methods. Ligament footprints in the humerus and the ulna were registered. Ligament length and isometric points were defined. The locations of the isometric points were imported into both methods to be compared.

**Results:**

There was no significant difference between 2 methods in calculating the length in every zone. There was also no significant difference in determining isometric ligament's origin point, which is located approximately 18.2 ± 4.0 mm and 18.4 ± 2.9 mm for single and multiple CT, respectively, measured inferolaterally from medial epicondyle.

**Conclusions:**

A solid preoperative plan is critical when predicting tunnel locations due to the difficulty in finding isometric points and the individuality of optimal bone tunnel locations. Using single CT scan, optimal locations can be predicted with the same accuracy as a multiple CT scans with less radiation exposure.

## 1. Introduction

Various studies have reported the anterior oblique band of the medial collateral ligament (MCL) of the elbow joint to be the primary stabilizer of valgus stress [1–4]. MCL function is vital for overhead athletes such as baseball pitchers. These throwing athletes frequently develop MCL insufficiency due to the repetitive valgus stress applied to their elbows during the throwing motion.

Since the first successful MCL reconstruction was performed in 1974 by Jobe et al. [5], various attempts have been made to determine the optimal bone tunnel positioning in patients with MCL injury. A previous study suggested that the isometric portions of the MCL's anterior bundle are the optimal locations for bone tunnels [6]. However, intraoperative determination of exact isometric points is often challenging, leading to difficulties in reconstruction.

A recent 3-dimensional (3D) study described a method for predicting the isometric points within the MCL's anterior bundle [7]. However, this method requires multiple computed tomography (CT) scans and the abovementioned study investigated CT scans of elbows at five different flexion angles: 0°, 40°, 80°, 120°, and 135°. In a clinical daily setting, the single CT scans will become convenient and practical for both health care provider and patient. The benefit of being less time consuming and having less radiation exposure to the patient will be highly valued.

To our knowledge, no study has been done to assess the capability of single CT scan to predict isometric points. Hence, the present study aimed to develop an applicable and reliable preoperative method for identifying the isometric points for MCL reconstruction by using single 3D CT modeling. We hypothesized that single CT scan could replace multiple CT scans with comparable accuracy by overcoming its higher exposure to radiation.

## 2. Materials and Methods

Following Institutional Review Board approval (ethical review number 2015-039), the elbows of five male volunteers (mean age: 28, range: 26–31) with no history of evident trauma of the upper extremity were CT scanned. Each elbow was scanned four times at 0°, 45°, 90°, and 135° of elbow flexion in neutral rotation as baseline data. Elbow positions were maintained with a custom-made plastic frame and nonmetal fixator. The images were saved as digital imaging and communications in medicine (DICOM) files. All the acquired images were subsequently exported to computer-aided design (CAD) software (Mimics 18.0; Materialise, Leuven, Belgium) for 3D model rendering. These 3D models were then analyzed using two different approaches: (1) single CT and (2) multiple CT method.

## 3. Single CT Method

**Figure 1:**
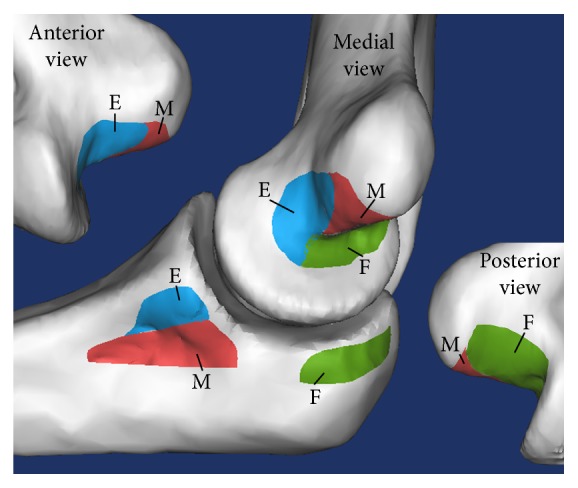
Registrations of MCL footprint in humerus and ulna. The footprint was divided into three zones: extension (E, blue), intermediate (M, red), and flexion (F, green).

The single CT method required ligament footprint and rotational axis data. By using 3D models rendered in full extension, humeral and ulnar anatomical footprint areas were registered for each 3D model (Figure 1). These locations were based on a previous study that described anatomy of MCL's footprints. In this fundamental MCL's footprints study, the author described the ligament topography which consists of extension (E), intermediate (M), and flexion (F) zones [8]. The footprints were then exported into a cloud of 3D vertices, comprising thousands of points on each area [9]. The ligaments were defined as the connection between a single vertex point at the humeral origin and a single point at the ulnar insertion on their respective areas.

The ulnohumeral joint axis was defined by the best-fit line through the center of all best-fitting circles in transverse cross-sections within the distal humerus. The best-fit line was obtained using the least squares method at the geometric centers of the capitellum and trochlear groove. This algorithm was previously validated by research that reported an intraclass correlation of 0.9 for both intra- and interobserver reliability [10].

The three-dimensional point cloud of ligament footprint and rotational axis was then imported into MATLAB software (MATLAB 2013b; MathWorks, Inc., Natick, Massachusetts, United States) for mathematical analysis of elbow movement and pairing of each ligament for length measurement. Pairing controlled execution program based on the standard mathematical combination algorithm was written prior to elbow's movement simulation. Elbow movement was simulated from full extension (0°) to full flexion (135°) through 1° increments.

## 4. Multiple CT Method

The 3D ulnohumeral joints at 45°, 90°, and 135° were superimposed onto humeral and ulnar anatomical landmarks in the full extension (0°) model using a voxel-based registration technique [11, 12]. With the same footprints and vertices as in the single CT method, the length of ligaments for each area at every static flexion angle (0°, 45°, 90°, and 135°) was calculated.

## 5. Ligament Length and Isometric Point

**Figure 2:**
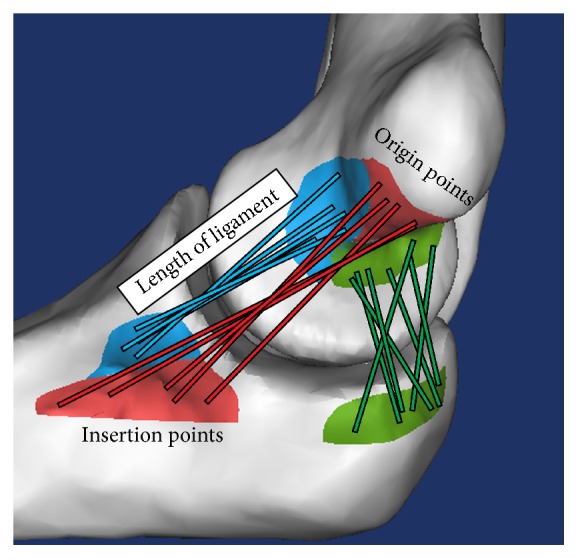
Representation of the connection and length measurement of ligaments from origin areas to their respective insertion areas.

**Figure 3:**
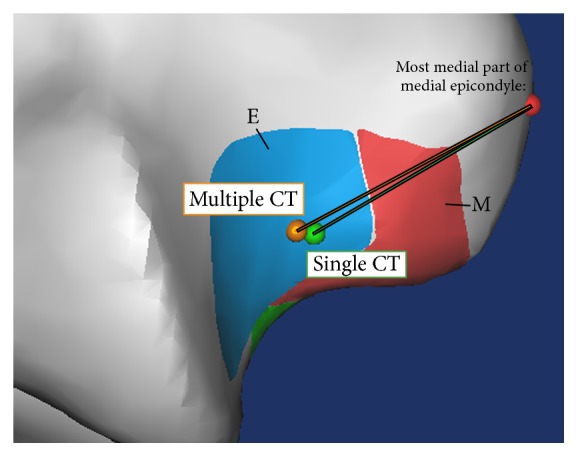
Isometric ligament origin locations relative to the medial epicondyle (red ball) after being analyzed with single CT (green ball) and multiple CT scan methods (orange ball).

The ligament length was measured from the origin to its insertion point in each footprint area (Figure 2). Isometric points were defined using the ligament length with the smallest variance (VAR < 0.1 mm) in each method. These isometric points were then imported back into the CT scan models to determine their location on the bone surface. The location of the isometric points was measured by using the most medial portion of the medial epicondyle as the reference landmark (Figure 3).

## 6. Statistical Analysis

To verify the accuracy of the single CT method, a paired Student's* t*-test was used to analyze whether (1) there was any significant difference between single and multiple CT scan methods in measuring the length of ligaments in each footprint area and (2) there was any significant difference in the location of isometric points found by the single and multiple CT scan methods. The significance level of the test (*P* value) was set to 0.05.

## 7. Results

**Figure 4:**
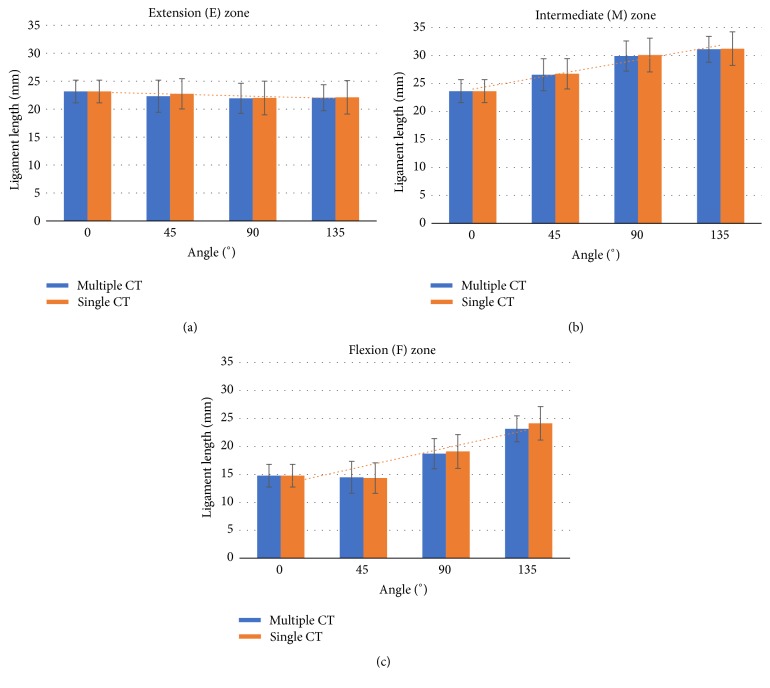
Result of length measurement using single and multiple CT method in extension (a), intermediate (b), and flexion zone (c). The higher the length of the ligament, the more taut the ligament.

The single CT method found no significant difference in ligament lengths compared with those determined by multiple CT scans in E, M, and F zones (*P* = 0.29, 0.30, and 0.28, resp.) (Figure 4). The average error of the single CT scan method compared with multiple CT scan in terms of ligament length was less than 1.5 mm in all footprint areas (1.29 ± 0.75 mm for E zone, 1.42 ± 0.81 mm for M zone, and 1.05 ± 0.80 mm for F zone) (Table 1).

There was also no significant difference in determining the location of the isometric points between the two methods (*P* = 0.37 and *P* = 0.38, resp.) (Table 2). The humeral isometric point calculated by single CT was located approximately 18.2 ± 4.0 mm (range, 14–24 mm) inferolateral from the medial epicondyle, whereas the ulnar side isometric insertion point was located approximately 32.2 ± 3.3 mm (range, 30–37 mm) distal from the medial epicondyle. The multiple CT method yielded similar results; the distances to the isometric ligament's origin and insertion measured 18.4 ± 2.9 mm and 31.9 ± 4.4 mm, respectively.

The average length of isometric ligaments calculated by single CT was similar to that by multiple CT (21.76 ± 1.4 mm and 21.35 ± 1.7 mm, resp.). Both of these results showed that the accuracy of single CT was comparable to that of the multiple CT method.

## 8. Discussion

Our study proved that single CT scan should replace multiple CT scans in determining optimal locations for bone tunneling for MCL reconstruction for two reasons. First, the accuracy of this practical method was found to be comparable to that of the multiple CT scans method, which is considered the gold standard for determining the isometric points within the human MCL. [7] Ligament length measured by single CT scan in E, M, and F zones showed no significant difference from the length measured by multiple CT scans in their respective areas, with less than 1.5 mm in error. Secondly, this method is applicable to a clinical setting as it requires simulation and modeling data from only one session of 3D CT scan, which means less harm by means of less radiation exposure for the patient and being less time consuming compared with a multiple CT scans session.

Our study showed that ligament origins are found near the rotational axis and mostly found in the extension (E) zone (Figure 3) when the ligament with the smallest changes in length (0 < VAR < 0.1 mm) was considered the most isometric ligament. However, several studies have previously shown that the isometric ligament origin can be found in a similar location to the guiding bundles' origin [8], which is at the intersection of the ligament footprint zones. Similar to a previous study, we could not find a true isometric ligament (VAR = 0) in the MCL [13]. Nonetheless, we found in our present analysis that the optimal humeral and ulnar tunnels were located approximately 18.4 ± 2.9 mm and 31.9 ± 4.4 mm, respectively, from the medial epicondyle. This has the potential to improve the results of MCL reconstruction, especially the single-strand ligament reconstruction technique [14].

There were some limitations to this study. First, our study is limited by the small number enrolled. Previous study also has similar limitation regarding sample size [12]. However, such pilot study does not necessarily follow the traditional statistical approaches because the conventional power analysis is crude owing to the small sample size. Secondly, we analyzed normal elbow joints with neutral rotation only. As this is our pilot study, such normal baseline value will serve as pertinent data towards future study. A future study involving 3D modeling of patients with MCL injury or pronated elbow would be opportune to evaluate the validity of our method, although we speculate that it is applicable as long as the patient has no dislocation of the elbow joint. The ulnar and humeral footprints were manually registered based on the anatomic studies and surgeon's experience and knowledge because all of the 3D models analyzed in this study were rendered from CT images. Lastly, we did not consider the angulation of the tunnel, which should be considered in MCL reconstruction surgery, in addition to the tunnel locations [15]. However, the value of this study is that by applying a single CT scan we are able to find isometric tunnels for MCL reconstruction comparable with the results of the previously described multiple CT scans method.

## 9. Conclusion

In conclusion, due to the difficulty in finding isometric points and the individuality of the optimal bone tunnel locations, a solid preoperative plan for predicting the optimal bone tunnel locations in patients with MCL injury is critical. Using a single 3D CT scan modeling method, optimal locations can be predicted in a clinical setting with the same accuracy as the multiple CT scans method with less radiation exposure.

## Figures and Tables

**Table 1:** Ligament length measurement error of single CT compared to that of the multiple CT method for each subject and position.

Subject	Extension (E) zone (mm)				Moderate (M) zone (mm)				Flexion (F) zone (mm)			
	0°	45°	90°	135°	0°	45°	90°	135°	0°	45°	90°	135°
1	0.00	1.48	1.88	1.95	0.00	1.70	2.22	2.30	0.00	0.77	1.58	1.77
2	0.00	0.21	0.36	0.39	0.00	0.15	0.11	0.58	0.00	0.51	0.16	0.58
3	0.00	1.17	1.13	2.64	0.00	1.68	1.99	2.64	0.00	0.05	1.69	2.30
4	0.00	0.36	0.79	1.40	0.00	0.61	0.76	1.20	0.00	0.10	1.04	1.25
5	0.00	1.90	2.23	1.46	0.00	1.37	2.02	1.96	0.00	0.57	0.75	2.62

Mean	0.00	**1.02**	**1.28**	**1.57**	0.00	**1.10**	**1.42**	**1.74**	0.00	**0.40**	**1.04**	**1.70**
SD	0.00	0.72	0.77	0.83	0.00	0.69	0.93	0.84	0.00	0.31	0.63	0.82

**Table 2:** Multiple CT and single CT-measured tunnel location of isometric ligament relative to the most medial part of the medial epicondyle.

Subjects	Multiple CT		Single CT	
	Origin location (mm)	Insertion location (mm)	Origin location (mm)	Insertion location (mm)
1	16.82	30.49	14.36	29.66
2	16.45	25.38	15.66	29.56
3	15.97	32.91	16.43	30.68
4	20.14	33.32	20.37	34.18
5	22.68	37.38	24.08	37.12

Mean	**18.41**	**31.90**	**18.18**	**32.24**
SD	2.895	4.405	3.989	3.309

## Additional Points

*Clinical Relevance*. It is practical preoperative planning for MCL reconstruction.* What Is Known about This Subject*. Multiple CT scan predicts the isometric tunnel placement for MCL reconstruction.* What This Study Adds to Existing Knowledge*. Our study details are to demonstrate a useful method for identifying isometric tunnel placement using a single computed tomography (CT) scan. The method itself is reproducible, applicable, accurate, and, to our knowledge, novel.
